# Genetics and Expression Analysis of Anthocyanin Accumulation in Curd Portion of Sicilian Purple to Facilitate Biofortification of Indian Cauliflower

**DOI:** 10.3389/fpls.2019.01766

**Published:** 2020-01-30

**Authors:** Shrawan Singh, Pritam Kalia, Rahul Kumar Meena, Manisha Mangal, Sabina Islam, Supradip Saha, Bhoopal S. Tomar

**Affiliations:** ^1^ Division of Vegetable Science, ICAR-Indian Agricultural Research Institute, New Delhi, India; ^2^ Division of Agricultural Chemicals, ICAR-Indian Agricultural Research Institute, New Delhi, India

**Keywords:** *Brassica oleracea* var. *botrytis*, *Brassica oleracea* var. *italica*, pigmentation, high performance liquid chromatography, RT-PCR, molecular markers

## Abstract

The present study was undertaken to know the genetics of purple color of cauliflower curds using a Sicilian purple ‘PC-1’ and a white curding mid-late group genotype of Indian cauliflower. For this, a cross was attempted between ‘DC-466’ (white curd) and ‘PC-1’ (purple curd) and observed intermediate level of purple pigmentation on curds in F_1_ plants. Segregation of F_2_ population (173) revealed that the purple color of the curd was governed by a single gene dominant over white, but the expression of trait was incomplete. It was substantiated by segregation of plants of BC_1_ and F_2:3(intermediate)_ generations into 1(white):1(intermediate) and 1(white):2(intermediate):1(intense), respectively. The F_2_, B_1_, and B_2_ generations segregated into purple(intermediate to intense): white curding plants in the ratio of 126: 47, 26:24, and 40:0, respectively fitting well with the Mendelian ratio of single gene for purple curds. However, purple pigmentation on curds ranged from very light to intense, which corroborated with the wide range of anthocyanin content in F_2_ (3.81–48.21 mg/100 g fw). Out of three molecular markers from high resolution map of *Pr* gene in purple color cauliflower ‘Graffiti’, only BoMYB3 marker could distinguish purple and white curding parents but did not show co-segregation while investigated in F_2_ population. Expression of *BoMYB1* gene was up regulated in both the purple curd genotypes ‘PC-1’ and ‘Graffiti’ in comparison to white curded ‘DC-466’, while *BoMYB2* gene was slightly upregulated in ‘PC-1’ but down regulated in ‘Graffiti’. Occurrence of ‘broccoli type’ F_2_ individuals and their genetic stability in F_2:3_ support the intermediate position of ‘Sicilian purple’ between broccoli (Calabrese) and cauliflower. There was not any correlation between curd coloration and pigmentation on apical leaf and stem portion, indicating difference of expression in ‘PC-1’ than ‘Graffiti’. The information obtained is useful for breeding anthocyanin rich attractive purple curding ‘specialty cauliflower’ for better consumer health and growers’ earnings.

## Introduction

Novel plants always attracted humans for health and nutrition purposes, and their acceptance remains high in case the regulating gene(s) are natural mutants. Purple color in plants is due to anthocyanins which have strong free radical scavenging activity and, thereby, good for health (Hwang et al., 2012). They belong to flavonoid group of polyphenol and impart red, purple, blue colors, and their different shades to plant parts to attract pollinators and consumers. They have biological functions in plants and human health due to metal chelation, positively charged oxanium, hydrogen donation, and protein binding properties (Kong et al., 2003). In plants, they protect chloroplast from photo-oxidative and photo-inhibitory damages caused by free radicals and ultra violet B light (Klaper et al., 1996) and for modification of captured light quality and quantity (Barker et al., 1996; Dodd et al., 1998). In human beings, anthocyanins help in reinforcing the critical balance between production and neutralizing reactive oxygen species (ROS) and reactive nitrogen species (RNS), thereby reducing the oxidative stress (Ozcan and Ogun, 2015). Preventive role of anthocyanins also reported against atherosclerosis, venous insufficiency, cardiovascular diseases, certain cancers, and other chronic diseases (Lila, 2004). *In vitro* studies further revealed inhibitory role of anthocyanins for activities of *COX-1* and *COX-2* genes which are involved in tumorigenesis (Hou et al., 2004a; Christian et al., 2006; Welch et al., 2008), induction of apoptosis of human promyelocytic leukemia (HL-60) cells due to specific ortho position of the hydroxyls in some anthocyanidins (Hou et al., 2003) and inhibition of activator protein 1 (AP-1) transcriptional activity and cell transformation (Hou et al., 2004b). Although, there is no specific recommended dietary guidelines for anthocyanins but the Chinese Nutrition Society (2013) proposed 50 mg/capita/day while FAO/WHO Expert Committee extended the specific proposed level to 2.5 mg/kg human body weight/day (for grape skin) (Wallace and Giusti, 2015). On the other side, the toxicity level of anthocyanins is also not observed in rats even up to 20 mg/kg body weight of rats and 25 mg/kg body weight of mice. Therefore, taking anthocyanin rich foods appears to be good for consumers’ satisfaction and health attributed by their intrinsic biological functions. More than 600 types of anthocyanins have been reported in plants but only six of them such as pelargonidin, cyanidin, peonidin, delphinidin, petunidin, and malvidin contribute 90% of anthocyanin present in the nature and 50% of them are acylated (added with acyl group, R-C = O).

**Figure 1:**
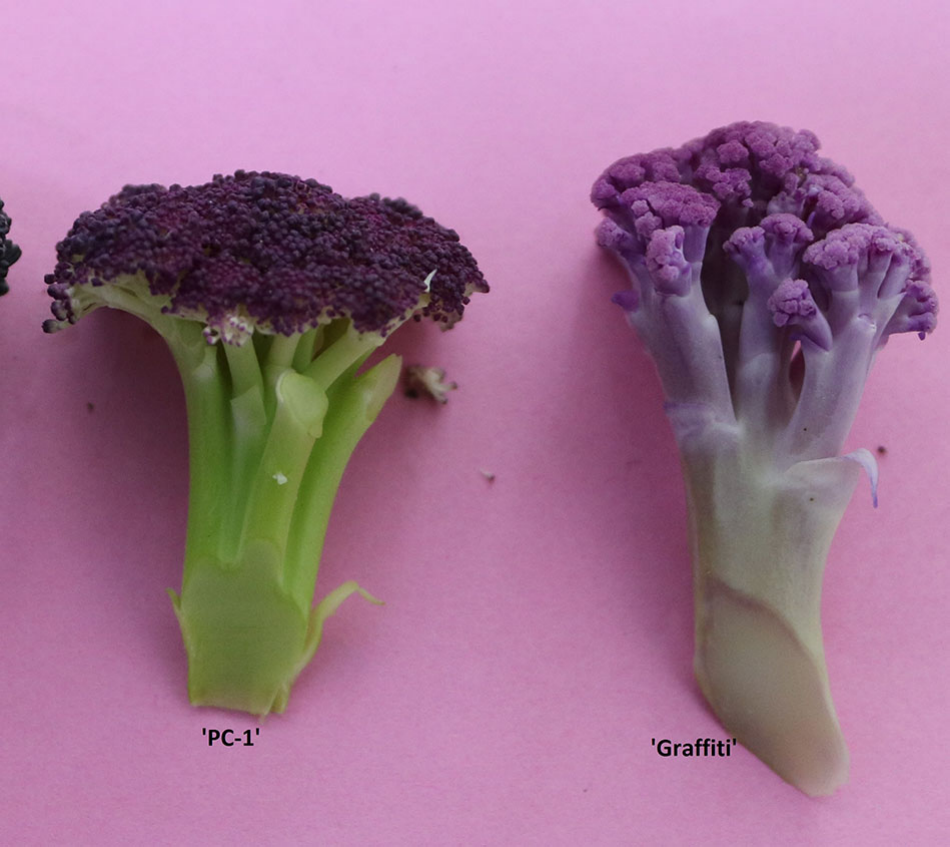
Anthocyanin pigmentation in curd fractions of ‘PC-1' and ‘Graffiti'.

Among the members of *Brassica oleracea* L., anthocyanin biosynthesis and accumulation occurs due to natural mutations as reported for purple cauliflower by Chiu et al. (2010).It has been genetically investigated for curd (pre-floral apical meristem) in purple cauliflower variety ‘Graffiti’ as a single semi-dominant gene *Pr* (Chiu et al., 2010), in ornamental kale as a single dominant gene *BoPr* for purple leaf (Liu et al., 2017), *BrPur* gene in Chinese cabbage (Wang et al., 2014), and similarly red leaf trait by a single dominant *Re* gene in ornamental kale (Ren et al., 2015). Genetic regulation of red leaf color in cabbage was indicated by Yuan et al. (2009) but nature of genetics has not been proposed yet. In cauliflower, the variety ‘Graffiti’ represents late or European group of cauliflower and obligatorily requires 10–16°C for curd initiation and development and 4–7°C for 5–6 weeks for vernalization. It produces typical ‘curd’, which is a modified pre-floral apical meristem. We could observe that it produces good quality curds during January end to February months in plains of North India, but bolting (elongation of flower stalk) and seed setting does not occur due to deficit of vernalization period. Alternatively, ‘Sicilian purple’ is botanically an intermediate of broccoli and cauliflower (Gray, 1982; Branca et al., 2018) and appears closer to broccoli. It forms curd-like heads at a temperature range of 16 to 25°C and does not require vernalization but needs cold temperature for bolting and flowering. Hence, it produces attractive full size cauliflower-like curds during December–March months, set seeds profusely, and is easily crossable with Indian cauliflower (Singh et al., 2019). Unlike ‘Graffiti’, it accumulates anthocyanin only in ‘pre-floral rudimentary buds’ (**Figure 1**) demonstrating the difference in genetic regulation of anthocyanin content between the two varieties. Although, Quiros and Farnham (2011) did mention the view of N. Acciarri that purple curd in ‘Sicilian purple’ governed by a recessive gene but we could observe intermediate type in F_1_ cross which contradicts the previous statement. Hence, it was required to investigate the genetics of purple curd in ‘Sicilian purple’ to plan its introgression in commercial varieties of broccoli and different maturity groups in cauliflower. Because, cauliflower and broccoli are important vegetable crops which are being grown worldwide on 1395152 ha with a production of 25984758 tons annually (FAOSTAT, 2017). It is 5^th^ important vegetable crop in India (which stand 2^nd^ after China) cultivated on 454,000 ha area with 8,557,000 tons production (NHB Database, 2017). It has large consumer base across the geographic regions and economic strata of Indian population and biofortifying this crop with health beneficial compounds like anthocyanins can benefit public sustainably.

Anthocyanin biosynthesis is a conserved pathway in plants and transcriptional regulation of three structural genes, namely R2R3MYB transcription factors, basic helix loop-helix (bHLH), and WD40 proteins appears to be the major mechanism to control this pathway (Broun, 2005). In *Arabidopsis*, accumulation of anthocyanins in vegetative tissues is mediated by four MYB proteins, namely PAP1 (Production of Anthocyanin Pigment1), PAP2, MYB113, and MYB114 and three bHLH proteins (Transparent Test8, Glabra3, and Enhancer of Glabra3) (Nesi et al., 2000; Gonzalez et al., 2008). As MYB transcription factor play an important role in accumulation of anthocyanins by regulating the transcription of structural genes. The *Pr* gene is a natural mutant in cauliflower itself and encodes for R2R3MYB transcription factor that exhibits tissue specific expression (Chiu et al., 2010). They indicated that Harbinger DNA transposon insertion in the upstream regulatory region of *Pr-D* gene governs the changes in anthocyanin accumulation in cauliflower ‘Graffiti’. But there is variation for (i) sink parts such as flowers, leaves, fruits, and stem among the botanical species and/or varieties, (ii) kind of predominant anthocyanins, and (iii) content value across the species and genotypes emphasize for case by case genetic studies. In some crops, such as *Arabidopsis*, the MYB factors require a bHLH in regulating anthocyanin biosynthesis (Gonzalez et al., 2008) while in crops like maize, each of them can act alone to activate transcription (Grotewold et al., 2000).

Identification of molecular markers is essential to handle the breeding materials for this environmental sensitive semi-dominant trait during introgression process (Yuan et al., 2009). They designed 17 gene-specific primers using highly homologous sequences in *B. oleracea* genome with *Arabidopsis* anthocyanin genomic information, all of them distinguished purple and green types. These markers were used by Chiu et al. (2010), and they isolated the *Pr* gene from cultivar ‘Graffiti’ through candidate gene-fine mapping analysis and developed a high resolution genetic map with three PCR-based markers, namely BoMYB2, BoMYB3, and BoMYB4 (<0.2 cM) linked to *Pr* gene. The cauliflower (*B. oleracea* var. *botrytis*), cabbage (*B. oleracea* var. *capitata*), broccoli (*B. oleracea* var. *italica*), and kale (*B. oleracea* var. *acephala*) are different botanical varieties of *B. oleracea*, hence chances of cross-transferability of markers is expected to be high. But, due to difference in evolutionary levels, validation of markers in Sicilian purple type was essential to use them in marker assisted introgression scheme.

Nutritionally enriched varieties with different nutrients and colors have promise, both for farmers to get premium price and for consumers as health food (Birol et al., 2015). The exotic ‘purple cauliflower’ material, although, remains suitable to grow for availability for only a short period, presently from December to January months, but it can be extended to mid-March (Singh et al., 2019). However, there is a huge demand for cauliflower both from growers’ and consumers’ for the entire rest of the year (Kalia et al., 2016) highlighting the need to develop genotypes for other maturity groups. With this background, the study was undertaken to (i) investigate genetics of purple color curds and anthocyanin content in Sicilian purple derived line ‘PC-1’ using Indian cauliflower as background and, (ii) validate the available linked markers from high resolution map of *Pr* gene in ‘Graffiti’ variety of purple cauliflower by molecular and gene expression studies.

## Materials and Methods

### Breeding Materials and Growing Conditions

**Figure 2:**
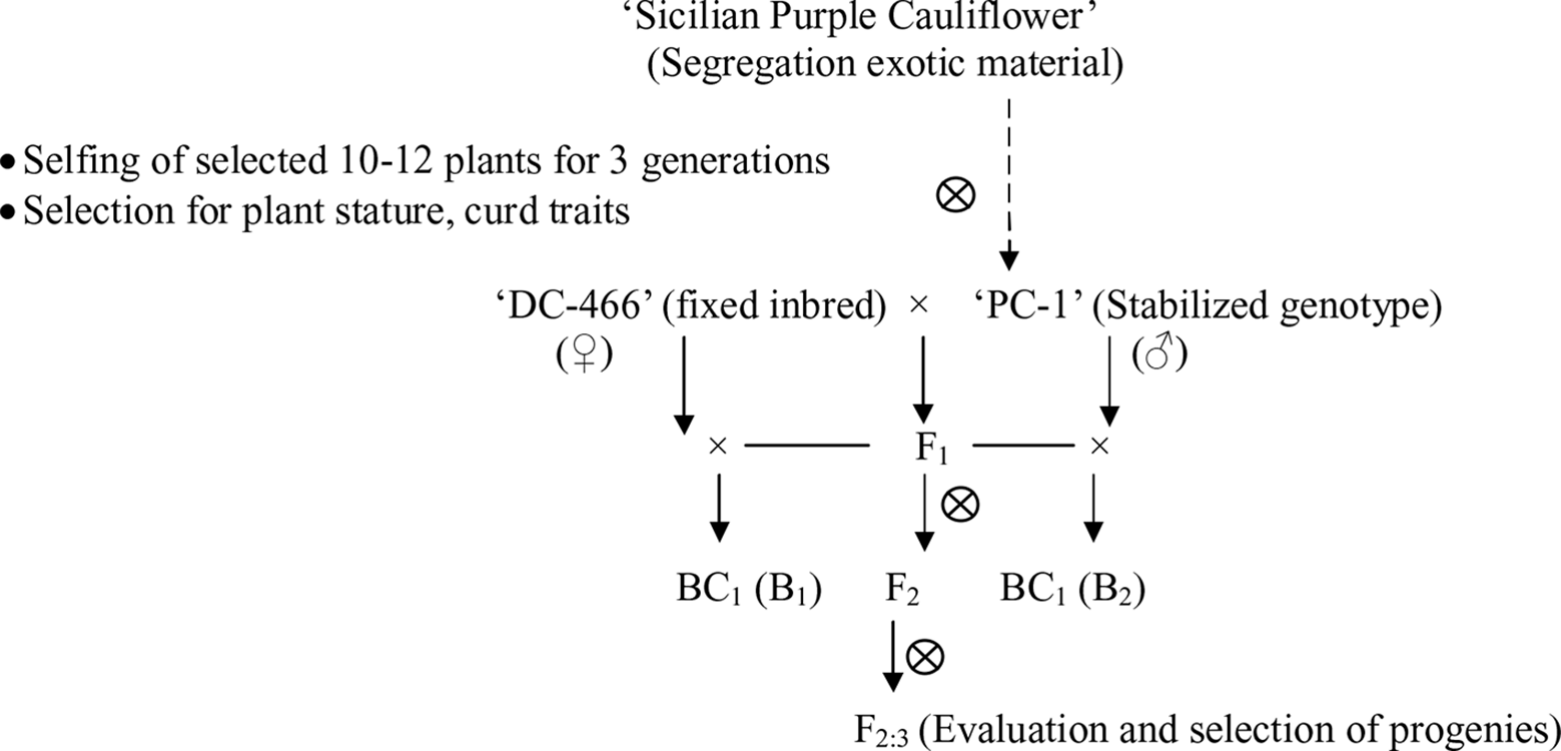
Population development procedure used in the study.

In present study, we used a purple cauliflower genotype ‘PC-1’ derived by recurrent breeding from ‘Sicilian purple’ at the Division of Vegetable Science, ICAR- IARI, New Delhi. Unlike ‘Graffiti’, it accumulated anthocyanin only in pre-floral buds (which constitute curds or heads) and not in stalk which retain attractive light green color holding, thereby, a unique purple-light green color combination to attract consumers’ preference. The breeding material for the study was generated by a breeding procedure depicted in **Figure 2**. In this, the ‘PC-1’ was crossed with ‘DC-466’, a white curding genotype of mid-late group of Indian cauliflower. F_1_ plants (28) from this were raised and two plants selfed by bud-pollination (2–3 days prior to anthesis) to generate F_2_ and were simultaneously backcrossed with parent genotypes ‘DC-466’ and ‘PC-1’ to develop B_1_ and B_2_ generations, respectively (**Figure 2**). The research farm is located in ICAR-IARI, New Delhi which has 28.63 N altitude and 77.15 E longitude with mean sea level being 228.61 meters. Mean monthly temperature during crop growing months i.e. October, November, December, and January were 26.3°C, 20.8°C, 15.7°C, 14.3°C, and 16.8°C, respectively.

All the populations F_2_, B_1_, B_2_, and F_1_ along with parents were sown in nursery beds (3.0 × 0.45 × 0.20 m) during September 2017 and 30 days old seedlings were transplanted on raised beds (≈15 cm) during October, 2017 at a spacing of 60 × 45 cm and the standard crop growing practices were followed as described by Singh and Sharma (2003). Well decomposed farm yard manure (FYM), nitrogen, phosphorus, and potash @ 25 t, 100 kg, 60 kg, and 50 kg per hectare, respectively were applied to the crop. Adequate soil moisture was maintained by regular irrigation at 7–10 days interval for proper crop growth and to avoid moisture stress which otherwise influence intensity of pigmentation.

**Figure 3:**
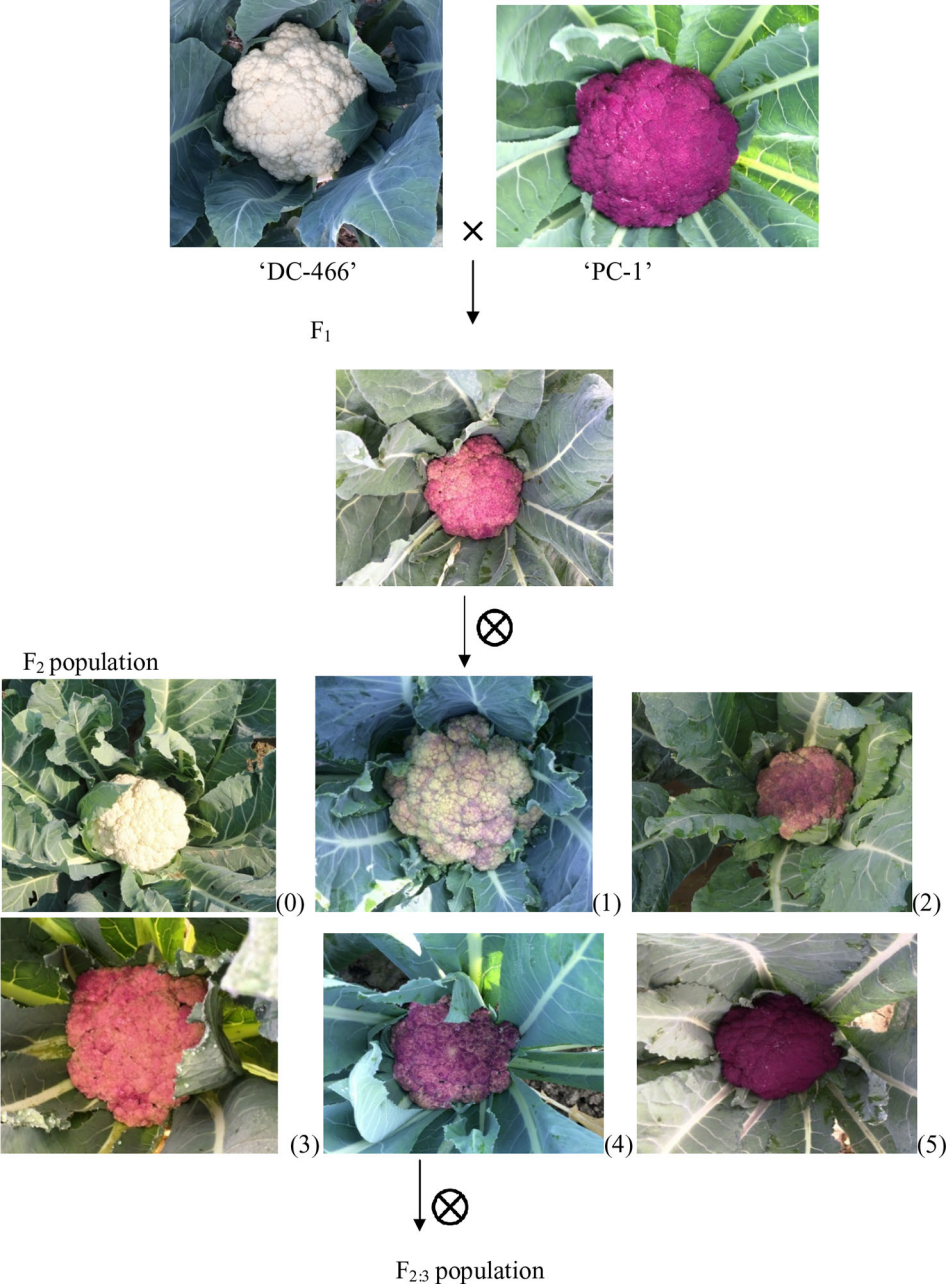
Observed curd color phenotypes of parents, F_1_, and F_2_ population. Curd color scale of ‘0–5'; 0 = white, 1–4 = light to intermediate purple; 5 = intense purple.

Curd traits were observed from each individual of F_2_, B_1_, B_2_, and F_1_ and parents using standard rulers and digital weighing balance. Curd color was observed through visual observation for presence or absence of purple pigmentation. Chiu et al. (2010) classified F_2_ plants into three categories *i.e.* white, light purple, and purple curds. We could, however, see a range of curd pigmentation in the segregating material. Hence, a phenotyping scale for purple curd trait was devised with ‘0–5’ score wherein, ‘0’—white, ‘1’—white base with slight purple dot-like pigmentation in parts of curd, ‘2’—whitish curds with light purple pigmentation on entire curd, ‘3’—light purple curds devoid of white color appearance, ‘4’—medium purple, and ‘5’—intense purple (**Figure 3**). Royal Horticultural Society color chart of Purple Group was used for clarification. The plants were also scored for purple pigmentation on stem and apical portion and curds were categorized based on texture. Further, 103 F_2_ individuals including 94 purple (88 intermediate and six intense) and nine white curding were advanced to F_2:3_ generation which were evaluated for curd traits during September 2018 to January 2019 period. Crop growing conditions and evaluation process of F_2:3_ progenies were same as that of earlier generations.

### Anthocyanin Determination

The sampling for anthocyanin determination in curd portion was carried out between 9.30–10.30 AM from the marketable curds (20–21 days after curd initiation) by cutting 2–3 well grown knobs (independent segments of curd, 15–20 g) with sharp knife and brought to lab in ice-box. The sampling at 20–21 days after curd initiation was based on a pre-sampling investigation performed from 3 to 24 days after curd initiation in ‘PC-1’. Curd samples were taken from 8 to 10 plants in each of 103 F_2:3_ progenies and bulked to represent respective progeny for analysis.

Total anthocyanins content were determined by pH differential method (Fulecki and Francis, 1968) as described by Chiu et al. (2005) and Yuan et al. (2009) with minor modification. In brief, 1 g sample was grounded in pestle-mortar separately with 20 mL of pH 1.0 buffer (1.86 g KCl in 980 mL water, added ca. 6.3 mL HCl, made volume to 1 L with water) and 20 mL of pH 4.5 buffer (54.43 g CH_3_CO_2_Na·3H_2_O in 960 mL water, add ca 20 mL HCl, make volume to 1 L with water). Mixture was centrifuged at 12,000*g* for 15 min at 4°C in refrigerated centrifuge (Sigma-Aldrich). Supernatant was filtered through Whatman No. 1 filter paper and diluted to a standard volume (25 mL). Absorbance was recorded at 520 nm using spectrophotometer (Eppendorf, Hamburg, Germany). Samples were analyzed in duplicate and mean values were taken for analysis. Total anthocyanin content (TAC) in samples were expressed as cyaniding-3-glucoside (C3G) equivalent and calculated using the following equation: TAC (mg/100 g fw) = (Abs_510_ at pH 1.0 − Abs_510_ at pH 4.5) × 449.2/26,900 × dilution factor. Here, 449.2 is molecular weight of cyaniding-3-glucoside and 26,900 is molar extinction coefficient.

### HPLC Analysis of Anthocyanin

Identification of prominent anthocyanin in curd portion of purple cauliflower ‘PC-1’ and ‘Graffiti’ as reference sample was done by reverse phase-high performance liquid chromatography (RP-HPLC) as per the procedure described by Yuan et al. (2009). For this analysis, 0.5 mg sample was extracted thrice in 25 mL of methanol: water: acetic acid (85:15:05) by grinding to colorless state using pestle and mortar at room temperature under dark condition. The extract was centrifuged at 12,000*g* for 10 min at 4°C in refrigerated centrifuge. Supernatant was collected and filtered through Whatman No. 1 filter paper and concentrated to 1 mL volume by rotary evaporator. The extract was diluted with water and samples were analyzed using HPLC with a diode array detector (Waters, Milford, MA). Twenty microliters of the sample was injected into SB-C18 (4.6 × 250 mm, 5 µm) column (Agilent Technologies, Rising Sun, MD, USA) and eluted using a mobile phase consisting of solvent A (5% formic acid: 95% water) and solvent B (100% methanol) at a flow rate of 1.0 mL/min and detection was done at 520 nm for 40 min run time. The elution program was followed as described by Wu and Prior (2005). The major individual anthocyanin peak were putatively identified based on previous publications with mass spectrometric analysis (Wu and Prior, 2005; Yuan et al., 2009; Chiu et al., 2010).

### Molecular Markers and PCR Analysis

Genomic DNA from young leaf samples of parents, F_1_ and F_2_ plants was isolated using CTAB method of Saghai-Maroof et al. (1984). Quality and quantity of DNA from each sample was estimated using ethidium bromide staining on 0.8% agarose gels. From F_2_, two bulks were made by using 10 individuals of each white and intense purple curding group. Genomic DNA was also extracted from five white curding genotypes, namely Pusa Shukti (401), DC-402, Pusa Himjyoti (PHJ), Pusa Sharad (309), and Pusa Paushja (476) and two of broccoli genotypes (PS-Palam Samridhi and PB-Pusa purple broccoli) for initial screening of markers.

A total of nine molecular markers from Chiu et al. (2010) were got synthesized from Sigma Chemicals Co. USA (**Supplementary Table 1**). Of them, three PCR based markers, namely BoMYB2, BoMYB3, and BoMYB4 were depicted on high-resolution genetic resolution map of *Pr* gene which spanned within 0.2 cM. The PCR reaction volume was 10 µl consisting of 50 ng genomic DNA, 5 µl of 2X master mix (OnePCR™, GeneDireX^)^ and 1.0 µM of each primer (0.5 µl forward and reverse equally). The PCR conditions were 94°C for 0.5 min; 35 cycles of 94°C for 30 s, 56–60°C for 30 s, and 72°C for 1 min; and a final extension at 72°C for 10 min (Chiu et al., 2010). The PCR-amplified DNA products were resolved by submerged horizontal electrophoresis (BioRad, USA) at a constant voltage of 120 V for 3 h using 4% (w/v) agarose gel and visualized under UV light fluorescence using Gel documentation system (Alpha imager, Cell Biosciences, Santa Clara, CA). The PCR products from marker analysis were scored visually for presence or absence of bands and their sizes were determined by use of 50 bp reference marker.

### RNA Extraction and Expression Analysis of *BoMYB* Genes Using Q-PCR Analysis

Four *MYB* genes, namely *BoMYB1*, *BoMYB2*, *BoMYB3*, and *BoMYB4* were studied for their expression pattern in parents, DC-466(P1), ‘PC-1’ (P_2_), and ‘Graffiti’ (as reference). Already published primer sequences of these genes (Chiu et al., 2010) were used for the study. For this, curd samples were collected in morning hours in liquid N_2_ and stored in deep freezer (−80°C) until RNA isolation. Three biological replicates were collected from each line. Total RNA from each sample was extracted using Tri-Xtract (G Biosciences, Geno Technology, Inc. St. Louis USA) following manufacturer’s guidelines and quantified with a nano spectrophotometer. 1 µl of isolated RNA was loaded on a denaturing agarose gel to check its concentration and integrity. 2 µg of total RNA was reverse transcribed using Verso cDNA synthesis kit (Thermo Fisher Scientific, Inc.) following manufacturer’s instructions. Tenfold dilution of cDNA was made and 1 µl of diluted cDNA was used as a template for each qPCR reaction. For a 10 µl qRT-PCR reaction, 5 µl of 2XSYBR Green master mix (Applied Biosystem, CA,USA), 2 μl nuclease free water, 1 μl each of forward and reverse primer of desired gene (100 nm) and 1 μl of template DNA was used. qRT-PCR analysis was performed on LightCycler^®^ 96 Real-Time PCR (Roche Diagnostics Corporation, Indianapolis, USA). The Q-PCR program comprised of initial denaturation at 95°C for 120 s, 40 cycles of denaturation at 95°C for 30 s, annealing at 55°C for 60 s, and extension at 72°C for 30 s. For Q-PCR two technical replicates were used per biological replicate of each genotype. The ΔCt value of each target gene was normalized with internal control Bo18S. The ΔΔCt values were calculated taking ΔCt value of ‘DC-466’ as calibrator and were used to plot graph to study the relative expression of each gene in the genotypes.

### Statistical Analysis

Mean, range, and standard deviation for data from anthocyanin content in plant materials was analyzed by use of Microsoft Excel software. The genetic analyses of curd color, apical, and stem pigmentation and curd texture in F_2_ and F_2:3_ were done by chi-square test using MS Software. Correlation analysis between curd color, apical, and stem pigmentation was performed by online OPSTAT software.

## Results

### Anthocyanin Rich ‘PC-1’ Inbred Line

Recurrent selection was performed using an exotic purple cauliflower ‘Sicilian purple’ which resulted into a promising ‘purple cauliflower-1’ (or ‘PC-1’) genotype. It has erect bluish green leaves, purple pigmentation on apical shoot and forms intense purple circular curds of 850–940 g weight. Its maturity period coincides with mid-late group of Indian cauliflower, i.e. from December end to mid-January which can extend up to mid-March by shifting planting dates.

### *Pr* Gene Governs Purple Curds in Cauliflower

All the 28 plants from ‘DC-466’ × ‘PC-1’ F_1_ cross produced an intermediate color curd phenotype, mostly of cauliflower-like curd shape with light purple pigmentation (**Figure 3**) indicating incomplete dominance of purple curds. The F_2_ population (173 plants) showed the monogenic segregation ratio of 1:3 (χ^2^ = 0.93, P_1df__=_ 3.48) with 47 white: 126 purple (including intermediate) plants. In F_2_, the intermediate phenotype had a range from light to medium purple, however, they were distinct from typical white curd trait of ‘DC-466’, hence they were merged into purple category. Further, the 173 individual F_2_ plants segregated into six minor groups viz., white, white base with slight purple dot-like pigmentation in parts of curd, whitish curds with light purple pigmentation on entire curd, light purple curds devoid of white color appearance, medium purple, and intense purple depicted as ‘0–5’ score in **Figure 3**. The grouping of F_2_ plants resulted into three prominent classes of curd color such as white (47): purple_(Light-medium)_ (95): purple_(Intense)_ (31), which segregated into 1:2:1 ratio (**Table 1**) substantiating the single locus governance of purple color of curd in source genotype. These results suggests that the purple color of curd in Sicilian purple derived genotype ‘PC-1’ is controlled by a single incomplete dominant gene. It was supported by the segregation of backcrosses *i.e.* B_1_ (F_1_ × DC-466) and B_2_ (F_1_ × PC-1), which resulted into prominent classes of curd color such as 24 white: 26 purple_(light-medium)_ and 0 purple_(dark)_ and 0 white: 23 purple_(light-medium)_: 17 purple_(dark)_, respectively.

**Table 1:** Segregation of F_2_ and backcross population for curd color trait in white: intermediate: purple categories.

Genotype	Total plants	White	Purple		Expected phenotypic ratio	χ2 value	*P* value
			Light-medium	Intense			
‘DC-466'	30	30	0	0			
‘PC-1'	38	0	0	38			
F_1_	28	0	28	0			
B_1_ (×'DC-466')	50	24	26	0	1:1	1.09	3.84
B_2_ (×'PC-1')	40	0	23	17			
F_2_	173	47	95	31	1:3 (1:2:1)	0.93 (4.92)	3.84 (5.99)

In parentheses, chi-square value for 1:2:1 ratio at P_0.05__at_ _2df_.

### Segregation of F_2:3_ Progenies

In total, 103 F_2:3_ progenies derived from 88 F_2_ individuals with intermediate curd color, nine with white curding F_2_ plants, and six with intense purple color were evaluated and the results obtained are presented in **Table 2**. There was no segregation in white and intense purple color progenies while all of the 88 intermediate purple progenies showed segregation for curd color. There were 1,144 plants from 88 progenies which segregated into 253 (white): 604 (light to intermediate purple): 287 (intense purple) fitting well with 1:2:1 ratio (χ^2^^=^ 5.60; *P*_2df_ = 5.99).

**Table 2:** Segregation of F_2:3_ progenies for curd color trait in white:intermediate:purple categories.

Genotype	No. of progenies	Plants in each progeny	No. of total plants	White	Purple		Expected Phenotypic ratio	χ^2^ value
					Light-medium	Intense		
F_2:3_ (from Intermediate F_2_s)	88	11–18	1,144	253	604	287	1:2:1	5.60 (*P*_0.05__at_ _2df__=_ 5.99)
F_2:3_ (from Intense purple F_2_s)	6	12–15	71	0	0	71	0:4	
F_2:3_ (from white F_2_s)	9	8– 13	84	84	0	0	4:0	

### Occurrence of ‘Broccoli Type’ Plants

**Figure 4:**
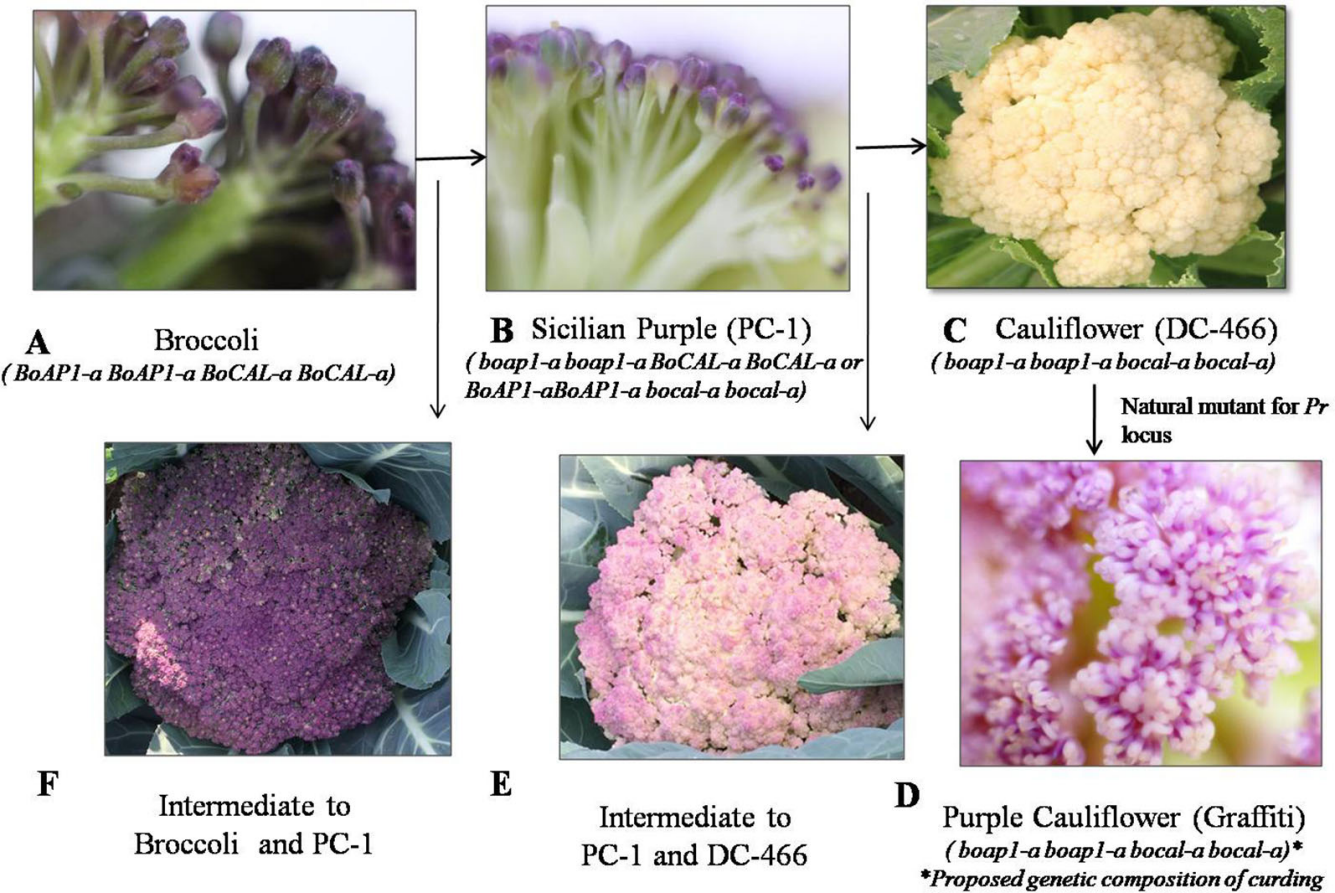
Flower buds in knob texture of curd/heads. Broccoli type **(A)**, Sicilian purple ‘PC-1’ type **(B)** white cauliflower ‘DC-466’ **(C)**, purple cauliflower ‘Graffiti’, **(D)** intermediate of ‘PC-1’ and ‘DC-466’ **(E)**, Intermediate of ‘PC-1’ and broccoli **(F)**.

We investigated 143 individuals of the F_2_ population from cauliflower cross ‘DC-466’ × Sicilian purple ‘PC-1’ for curd texture which segregated into 30 ‘PC-1’ type, 80 Intermediate type, 33 ‘DC-466’ type (**Figures 4A–F**). A clear distinction between all three broad types was observed for appearance and size of buds at maturity stage. The bud size and texture of ‘PC-1’ was observed to be completely different from ‘PC-1’. Interestingly, in the ‘PC-1’ type plants, we also observed six plants having broccoli type heads with medium coarse buds and medium intensity of purple coloration (**Figure 4F**). There was no segregation in progeny of such an F_2_ plant ‘PC-466-1-76’ in F_2:3_ generation for head texture and color (**Supplementary Figures 1A, B**). However, the average size of heads (310.5 ± 15.2 g) was smaller than the broccoli (640.0 ± 20.4 g). Some plants also formed crude curd with precocious floral bud primordia across its surface prior to floral arrest during head formation.

### Apical and Stem Pigmentation Do Not Segregate With *Pr* Gene

**Figure 5:**
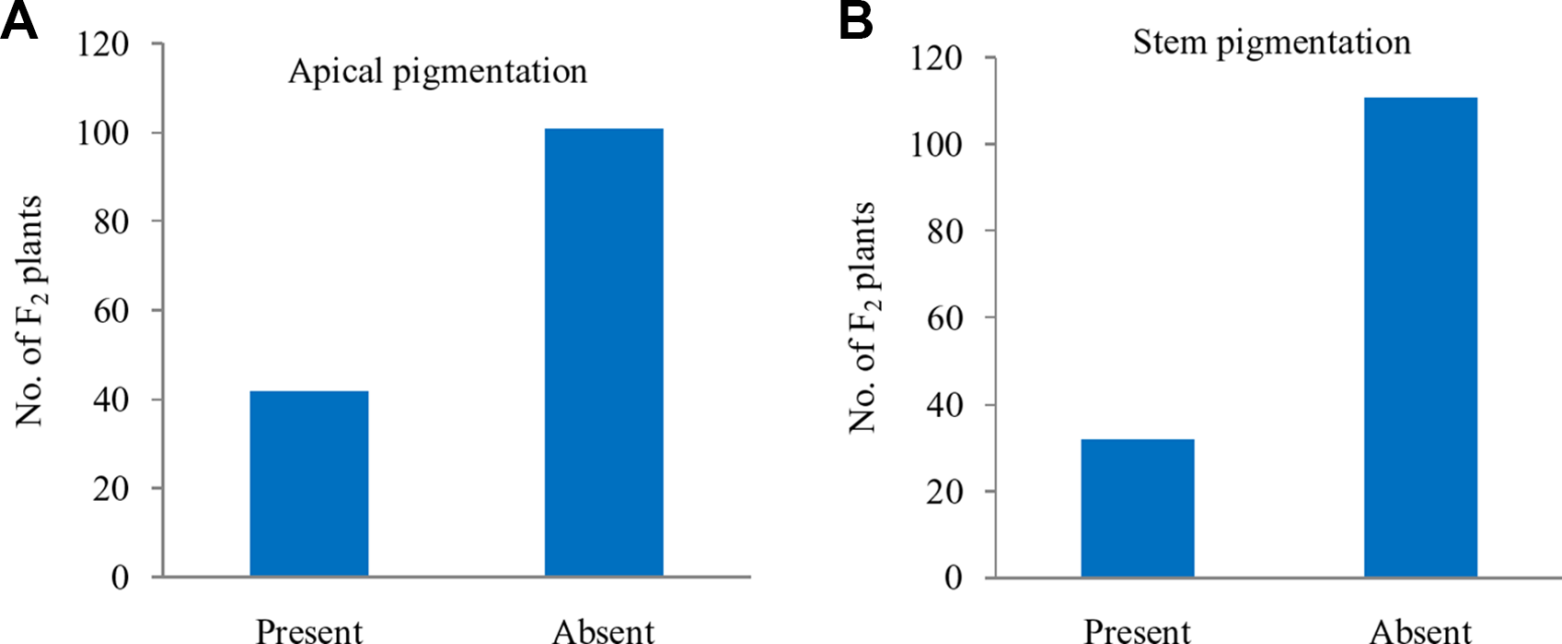
Segregation of F_2_ plants: apical pigmentation **(A)** and stem pigmentation **(B)**.

Further, these 143 F_2_ plants were observed for apical leaves for purple pigmentation and found that it was present in 42 plants while absent in 101 plants fitting well with the Mendelian ratio of single recessive gene (1:3 ratio; χ^2^ = 0.52; P_0.5 at 1df_ = 3.84) (**Figure 5A**). Similarly, the stem pigmentation also followed the same trend and plants segregated into 32 (present):111 (absent) plants (1:3 ratio; χ^2^ = 1.47; P_0.5 at 1df_ = 3.84) (**Figure 5B**). Contrasting pattern for segregation of purple curd phenotype from that of apical and stem pigmentation traits could be due to pigmentation in head portion of ‘PC-1’ unlike pigmentation in complete curd tissues in ‘Graffiti’ as shown in **Figure 1**.

### Marketable Stage Curds Contain Highest Anthocyanin

**Figure 6:**
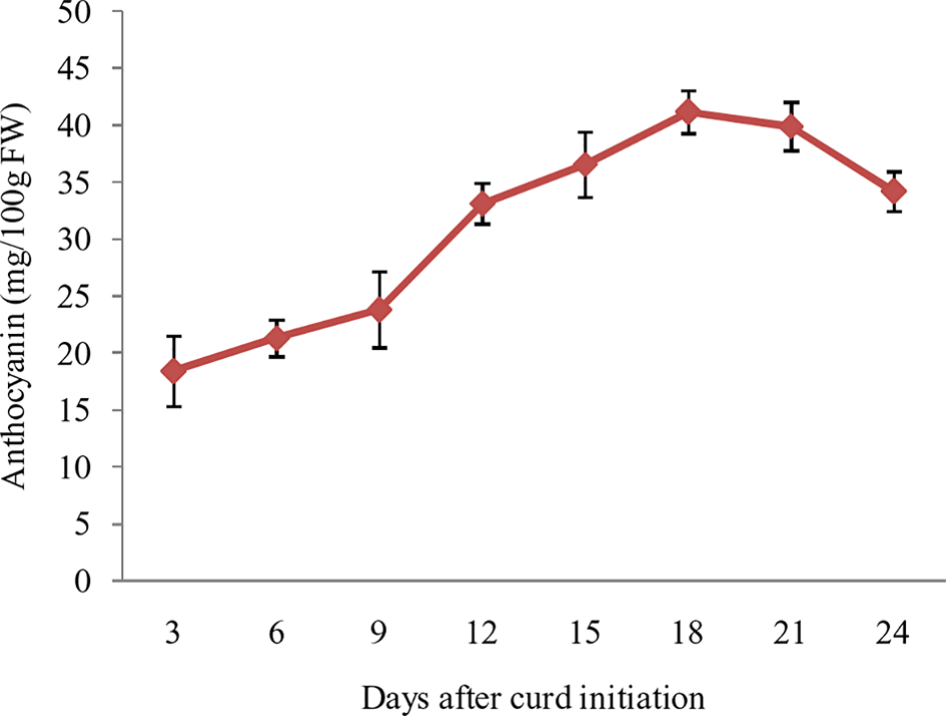
Anthocyanin content in ‘PC-1’ at different days after curd initiation.

The purple pigmentation in curd fraction of ‘PC-1’ was increased during curd development and attained most attractive color at full curd stage. However, we attempted a pre-sampling investigation by collecting small fractions of curds at different days *i.e.* 3, 6, 9, 12, 15, 18, 21, and 24 days after curd initiation in ‘PC-1’. The anthocyanin content showed increasing trend from 3^rd^ day (18.4 mg/100 g fw) to 21^st^ day (41.2 mg/100 g fw) of curd initiation, however, it declined afterward to 34.2 mg/100 g fw on 24^th^ day of curd initiation (**Figure 6**). This stage was corresponding to the compact and full size harvestable curds.

### HPLC Analysis of ‘PC-1’ for Anthocyanin Identification

**Figure 7:**
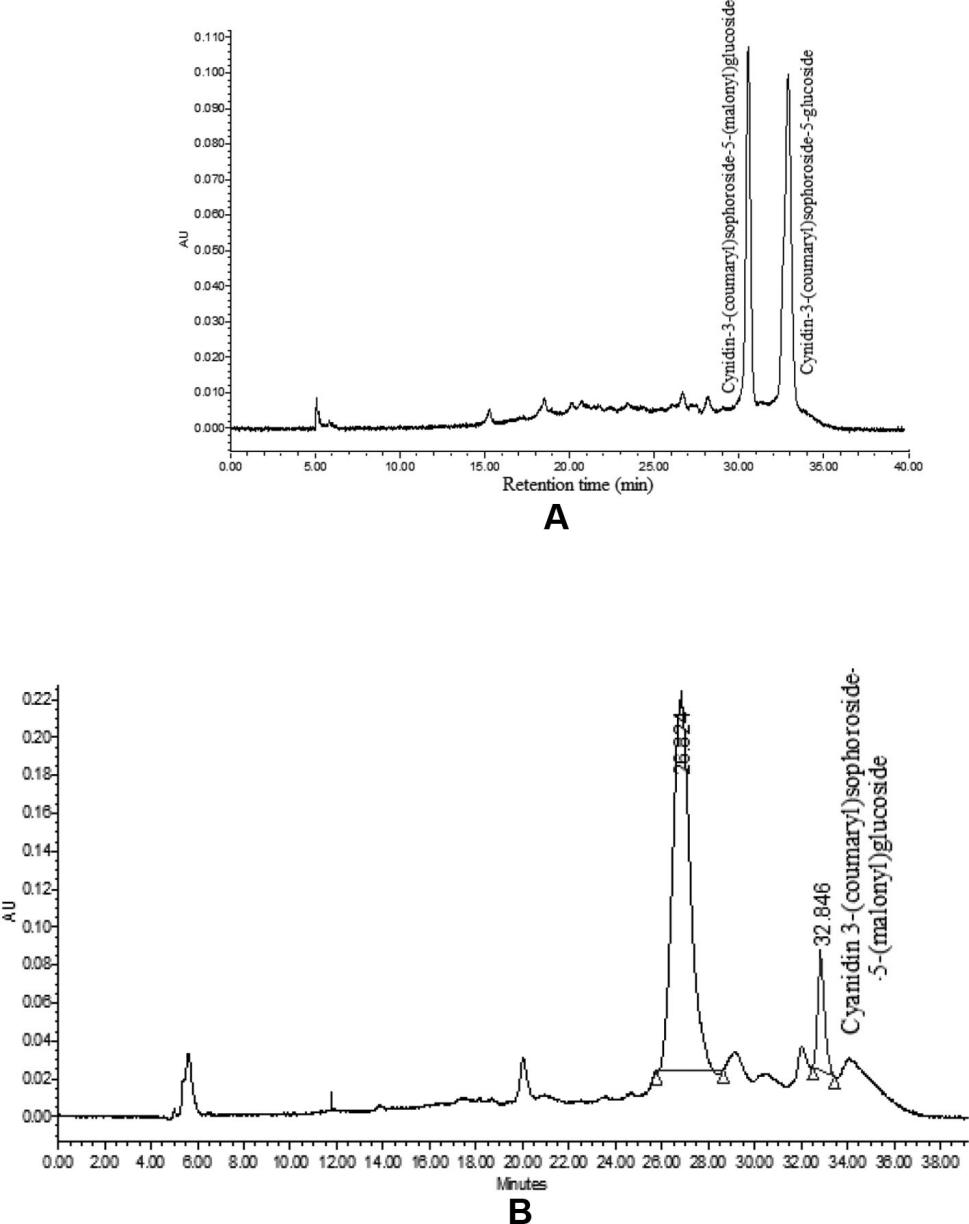
HPLC chromatogram of anthocyanin in ‘PC-1’ **(A)** and ‘Graffiti’ **(B)**.

The HPLC analysis of curd portion from ‘PC-1’ for anthocyanins indicated occurrence of only two prominent peaks as cyanidin-3-(coumaryl) sophoroside-5-(malonyl) glucoside and cyanidin-3-(coumaryl) sophoroside-5-glucoside (**Figure 7A**). We could observe six peaks for anthocyanins in curd samples of ‘Graffiti’ but only two of them were prominent (**Figure 7B**). The only peak for cyanidin 3-(coumaryl) sophoroside-5-(malonyl)glucoside (*t_R_*: 32.5 min) was found to be common in both ‘PC-1’ and ‘Graffiti’.

### Anthocyanin Content in F_2_ and F_2:3_ Populations

**Figure 8:**
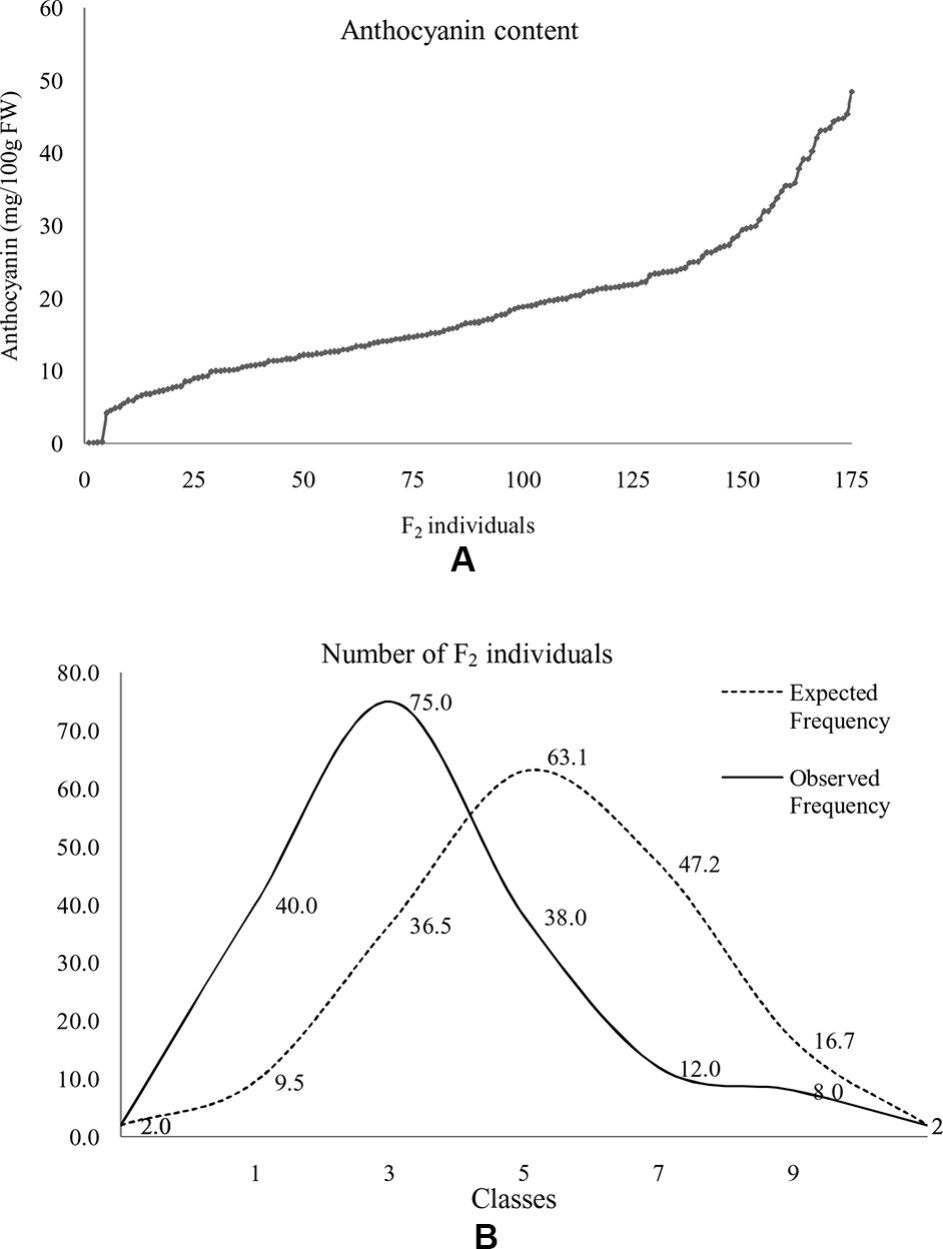
Distribution of anthocyanin content in F_2_ population. Anthocyanin in F_2_ plants **(A)** and distribution of F_2_ population with reference to normal distribution curve for anthocyanin content based on grouping of 10 mg/100 g FW series **(B)**. Here, 0 = < 1 mg or not detectable, 1 = 1–10 mg, 2 = 11–20 mg, 3 = 20–30 mg, 4 = 31–40 mg, and 5 = > 40 mg/100 g FW.

At horticultural maturity, the mean values of anthocyanin content in curds of ‘DC-466’ was observed to be <1 mg/100 g (0.29 ± 0.33 mg/100 g FW), while intense purple curds of ‘PC-1’ was found to contain 40.6 ± 2.74 mg/100 g FW. In F _1_ plants, it was observed to be 22.40 ± 4.51 mg/100 g FW which appears to be intermediate of both the parents. Anthocyanin content ranged from 0.05 to 48.21 mg/100 g FW in 173 F_2_ plants with mean value of 18.50 ± 9.98 mg/100 g FW (**Table 3**; **Figure 8A**). Out of them, 40 plants had <10 mg/100 g FW, and eight plants were observed to contain anthocyanin >40 mg/100 g FW. Based on anthocyanin content, we could categorize F_2_ population into six different groups (at interval of 10 mg/100 g FW) and results are presented in **Figure 8B**. The distribution of F_2_ plants for anthocyanin content didn’t follow the expected normal distribution pattern. Three plants had less than 1.0 mg/10 g FW anthocyanins. Maximum numbers of plants containing anthocyanins were found to be in 11–20 mg/g FW range (75 plants) followed by 1–10 mg/g FW (40 plants) while 38 plants were in 20–30 mg/100 g range, and only eight plants were found to be higher than 40 mg/100 g FW including ‘PC-1’.

**Table 3:** Anthocyanin content (mg/100 g FW) in parents, F_1_, F_2_, and F_2:3_ of ‘DC-466’ × ‘PC-1’ combination.

Genotype	No. of plants/progenies analyzed	Minimum	Maximum	Mean	STDEV
‘DC-466’	3	0.10	1.13	0.298	0.325
‘PC-1’	3	34.98	43.48	40.6	2.75
F_1_	2	18.23	29.58	22.4	4.51
F_2_	173	0.051	48.42	18.5	9.98
F_2:3(intermediate)_	88^*^	2.81	50.41	19.89	10.02
F_2:3__(intense_ _purple)_	6^*^	40.63	44.7	42.5	1.2
F_2:3(white)_	9^*^	0.1	2.09	0.7	0.46

*Bulk samples of progenies.

In F_2:3_ progenies, anthocyanin content was 42.50 ± 1.2 mg/100 g FW in six progenies of F_2_ (purple) and 0.70 ± 0.46 mg/100 g FW in nine progenies of F_2_ (white) (**Table 3**). The F_2_ population had 2/3 plants with purple (light to intense) curds while anthocyanin content distribution skewed toward the parent ‘DC-466’ (**Figure 8B**) suggesting incomplete dominance of mutant phenotype because only 31 plants had the intense purple phenotype. Interestingly, a few F_2_ plants (8) had anthocyanin content richer that than the ‘PC-1’ parent, and few (3) also had less anthocyanin than the ‘DC-466’ parent.

### Validation of Molecular Markers

The already reported nine molecular markers from high resolution map of *Pr* gene of purple cauliflower ‘Graffiti’ were first screened in genomic DNA of parents. Except BoMYB2m, all markers were amplified, however, only BoMYB3 could show polymorphism between white and purple curding parents (**Table 4**). Although, BoMYB2 marker did not produce amplicon in ‘Pusa Paushja’ (476)’ (a white curding genotype) but it failed to distinguish purple and white curding parents (**Supplementary Figure 2A**). These markers were also tested in five genotypes of white curding Indian cauliflower [Pusa Shukti (401), DC-402, Pusa Himjyoti (PHJ), Pusa Sharad (309), and Pusa Paushja (476)] and one each of purple broccoli [Pusa purple broccoli (PB)] and green broccoli [Palam Samridhi (PS)]. None of them discriminate between purple cauliflower/broccoli and white cauliflower/green broccoli (**Supplementary Figures 2A–E**). The BoMYB3 marker was further used for genotyping of 90 F_2_ plants, however, it did not show any association with *Pr* locus in ‘PC-1’ (**Supplementary Figure 3**).

**Table 4:** Screening of oligonucleotide primers in parents, bulk, and reference lines.

Primer	No. of amplicon	Amplicon size (bp)	‘DC-466’	‘PC-1’	Bulk (white)	Bulk (purple)	Green broccoli	Purple broccoli	309	PHJ	476	Polymorphism for purple curd
BoMYB1	One	130	+	+	+	+	+	+	+	+	+	Monomorphic
BoMYB2	One	331	+	+	+	+	+	+	+	+	−	Monomorphic
BoMYB3	One	110	−	+	−	+	−	−	−	−	−	Polymorphic
BoMYB4	One	264	+	+	+	+	+	+	+	+	−	Monomorphic
BoMYB2m	One	190	+	+	+	+	+	+	+	+	+	Monomorphic
BoMYB2m	−	−	−	−	−	−	−	−	−	−	−	No amplification
BoMYB3m	One	400	−	+	−	+	−	−	−	−	+	Monomorphic
BoMYB4m	One	110	+	+	+	+	+	+	+	+	+	Monomorphic
BoMYB4mR	One	130	+	+	+	+	+	+	+	+	+	Monomorphic

Amplicon present (+), absent (−).

### Expression Analysis of Anthocyanin Genes

**Figure 9:**
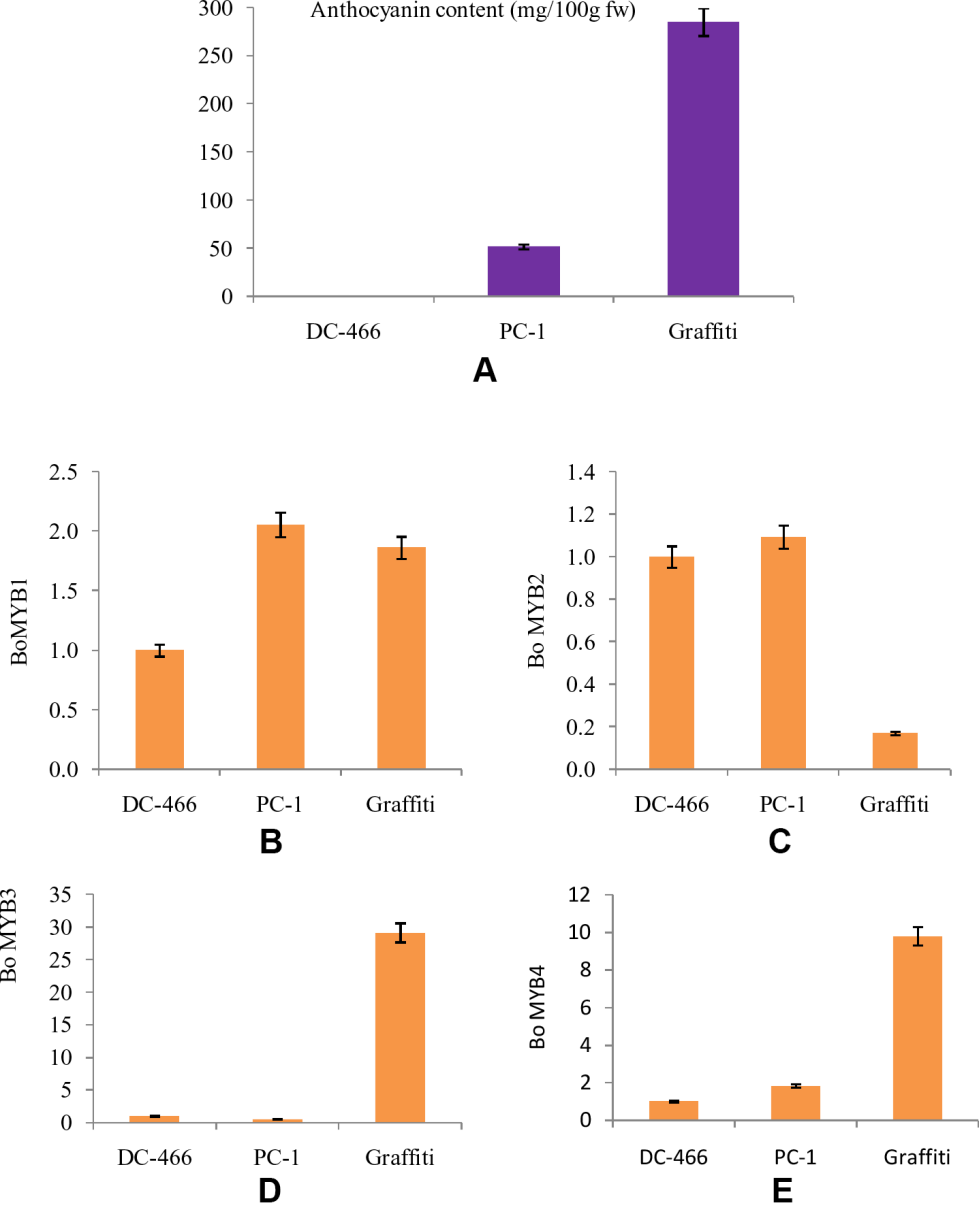
Anthocyanin content in test genotypes **(A)**, relative fold change in expression of genes, i.e. Bo MYB1 **(B)**, BoMYB2 **(C)**, BoMYB3 **(D)**, and BoMYB4 **(E)** in DC-466’, ‘PC-1’, and ‘Graffiti’.

The MYB transcription factors play an important role in accumulation of anthocyanins by regulating the transcription of structural genes. Hence, four *MYB* genes, namely *BoMYB1*, *BoMYB2*, *BoMYB3*, and *BoMYB4* were studied for their expression pattern in parents ‘DC-466’ and ‘PC-1’ along with ‘Graffiti’ as a reference and results are presented in **Figures 9A–E**. The expression of *BoMYB1* gene was up regulated in both the purple curding genotypes ‘PC-1’ and ‘Graffiti’ in comparison to ‘DC-466’. Although, its expression was approximately 0.20 fold lower in ‘Graffiti’ as compared to ‘PC-1’. The expression of *BoMYB* 2 gene was however, found to be slightly upregulated in ‘PC-1’ (by 0.09 fold) and downregulated by 0.83 fold in ‘Graffiti’. Both *BoMYB3* and *BoMYB4* genes were found to be substantially upregulated (29.14 and 9.80 fold, respectively) in ‘Graffiti’ while the former was down regulated in ‘PC-1’ by 0.47 fold, the latter, however, showed only 0.83 fold higher expression in ‘PC-1’ as compared to the white curding genotype ‘DC-466’.

## Discussion

‘Specialty cauliflowers’ or ‘rainbow cauliflowers’ or ‘colorful cauliflowers’ taste the same as the normal white varieties, but add splash of colors and nutrients to their food value. In fact, white or ‘bleached’ cauliflower contains only glucosinolates as a predominant functional constituent of health significance (Verkerk et al., 2009), while purple cauliflower provides anthocyanins additionally (Chiu et al., 2010). The new genotype ‘PC-1’ developed from exotic collection Sicilian purple produces attractive purple color curds during December to March months as well as flowers and sets seeds satisfactorily in northern plains of India during winter season. The ‘PC-1’ has purple pigmentation on apical and stem regions but intensity is low as compared to the ‘Graffiti’.

The segregation of F_2_ population was from white to intense purple, but it had a range from slight to intense pigmentation. Hence, we made three major classes *i.e.* white, light to medium purple, and intense purple curds for ease of interpretation of data. The observed values for segregation of F_2_ plants are in agreement with 1:3 for white: purple curds but they were following 1:2:1 ratio for white: intermediate: intense purple color similar to ‘Graffiti’ purple cauliflower reported by Chiu et al. (2010). Further, the segregation F_2:3_ progenies for curd color supported single semi-dominant gene for purple color curd trait. This indicates that the purple pigmentation in curd portion of cauliflower and head portion of Sicilian purple has similar genetic control despite variation in pigmented portion which is pre-floral apical meristem and pre-mature flower buds, respectively. However, the observations for purple pigmentation on apical shoot and stem parts in 143 F_2_ plants indicated for their recessive nature and single locus governance.

Occurrence of broccoli type plants in segregating F_2_ population could be due to influence of modifier genes on the degree and stage of inflorescence arrest and modification from the Indian cauliflower genotype ‘DC-466’. Unlike cauliflower curds which represents proliferation of arrested inflorescence and floral meristem (Carr and Irish, 1997), these plants produced broccoli-like heads with inflorescence spikes developed as a loose collection of immature flower buds. Distinct variation between buds of mature stage heads and curds in all three types could occur due to genetic factors. Although, occurrence of such plants is difficult to explain purely by ‘two gene theory’ of Smith and King (2000) for broccoli (Calabrese) to Sicilian purple to cauliflower domestication process, hence we believed that certain modifier genes for flowering trait (because broccoli heads are made up of immature flower buds) are playing a role in this phenomenon. The role of such genes in heading traits in *Brassicas* has already been suggested by Smith and King (2000). These modifier genes in Indian cauliflower might have evolved during its evolutionary process from European materials during past 200 years (*i.e*. after introduction from Europe in 1822) as a part of adaptive mechanism in cauliflower for sub-tropical Indian conditions. According to them, the Sicilian purple is an intermediate (*boap1-a boap1-a BoCAL-a BoCAL-a* or *BoAP1-aBoAP1-a bocal-a bocal-a*) type of Calabrese broccoli (*BoAP1-aBoAP1-a BoCAL-a BoCAL-a*) and cauliflower (*boap1-a boap1-a bocal-a bocal-a*). We purified the Sicilian purple exotic materials and fixed genotype ‘PC-1’ supposed to have *boap1-a boap1-a BoCAL-a BoCAL-a* or *BoAP1-aBoAP1-a bocal-a bocal-a* genes and with these gene combinations, it is difficult to get typical broccoli type plants (which should have *BoAP1-a BoAP1-a BoCAL-a BoCAL-a* in F_2_ but occurrence of heterozygous state of one of the two genes in ‘PC-1’ along with unknown tropical flowering genes and associated modifiers in Indian cauliflower might be leading to delay in inflorescence restriction stage from pre-floral apical meristem in curd (as in cauliflower) to immature floral buds (as in broccoli type plants). Hence, we propose the role of some other gene(s) in Indian cauliflower which led to occurrence of broccoli type plants in segregating materials from ‘DC-466’ × ‘PC-1’ combination, however it needs further investigation.

By HPLC analysis, we observed two prominent peaks for anthocyanins in curd samples of ‘PC-1’ and ‘Graffiti’. However, ‘Graffiti’ also had four additional peaks as reported earlier by Chiu et al. (2010) including cyanidin 3-(coumaryl-caffeyl) glucoside-5- (malonyl)glucoside as prominent peak along with minor peaks for cyanidin 3-sophoroside-5-glucoside (*t_R_*: 4.0 min), cyanidin 3-sophoroside-5-(malonyl)glucoside (*t_R_*: 8.2 min), cyanidin 3-(coumaryl)sophoroside-5-(malonyl)glucoside (*t_R_*: 32.5 min), cyanidin 3-(coumaryl)sophoroside-5-glucoside (*t_R_*: 43.6 min), and cyanidin 3-(ferulyl-caffeyl)sophoroside-5-(malonyl)glucoside (*t_R_*: 60.5 min) by use of HPLC electrospray ionization (ESI)-tandem mass spectrometry. Only cyanidin 3-(coumaryl) sophoroside-5-(malonyl) glucoside appears to be common in both the purple genotypes. The variations in number or peaks and anthocyanin content could be due to change in genotypes, expression of gene(s) depending upon sites of anthocyanin accumulation and laboratory instruments. In ‘PC-1’, anthocyanin is present only in pre-floral buds while in ‘Graffiti’, it expresses in pre-floral meristem and stalk portions.

In present study, the anthocyanin content were found to be less in segregating F_2_ population of ‘DC-466’ × ‘PC-1’ cross and parent genotype ‘PC-1’ in contrast to earlier reports of Chiu et al. (2010) in purple cauliflower variety ‘Graffiti’ (315 mg/100 g fw). This was mainly due to the genotype effect and also due to anthocyanin pigmentation site which appeared to be only pre-floral buds in ‘PC-1’ and pre-floral meristematic tissues in curd portion of ‘Graffiti’. However, high level of anthocyanin content in five F_2_ plants than the ‘PC-1’ indicated transgressive segregation for anthocyanin content due to modifier genes which might be involved in regulating anthocyanin biosynthesis or accumulation.

Scalzo et al. (2008) reported total anthocyanin content of 75.6 mg/100 g fw in red cabbage and 4.21 mg/100 g in violet cauliflower in addition to differences in pigment compositions of both the crops. Besides, anthocyanin in ‘PC-1’ was also less than that of red cabbage (50 to 182 mg/100 g fw) as reported by Yuan et al. (2009), which was mainly due to difference in pigmented tissues. Interestingly, anthocyanins from cabbage have strong antioxidant activity (22.0 mmol Fe(II)/g fw), which is approximately 10-fold higher than green cabbage (Yuan et al., 2009), suggesting for similar studies with purple cauliflower. Further, Jana et al. (2017) reported total anthocyanin content in red cabbage extract was found to be 86.004 ± 3.103 mg/100 g and elucidated the mechanism of ARCE mediated prevention of experimentally induced myocardial damage in rodent model. These investigations suggests for further studies to understand the potential of purple color *Brassica* vegetables for good health.

The molecular markers are useful tools for early stage detection of purple curding plants, otherwise we have to wait for curding stage to identify desirable plants in breeding materials. Notably, pigmentation in ‘PC-1’ gradually increases with curd maturity and reaches its peak at harvest maturity. Because of this, the curds of 18 to 21 days stage observed to have highest anthocyanin content, and it appears to follow sigmoidal kinetics in various plants as showed by Rogez et al. (2011) in acai fruits (*Euterpe oleracea*). To avoid this waiting period, the molecular markers (BoMYB2, BoMYB3, and BoMYB4), from a previously reported high resolution map for *Pr* gene in ‘Graffiti’ (Chiu et al., 2010), were used but they could not reproduce similar results in ‘PC-1’. However, BoMYB3 showed polymorphism between white and purple curding parents, but their segregation with purple curd didn’t show close association. Hence, we recommend for analysis of more number of markers to find linked markers. The polymorphism between two could be due to diverse nature of genetic constitution, because Sicilian purple and Indian cauliflower evolved in two different eco-geographical regions. BoMYB2 and BoMYB4 markers could not differentiate the white and purple curding parental genotypes as well. Further, these markers could not distinguish purple broccoli/Sicilian purple from white curding genotypes. Hence, it appears that (i) these markers are specific to purple cauliflower ‘Graffiti’ and (ii) source gene(s) for purple color in Sicilian purple is different from ‘Graffiti’. This was due to difference in genotypes because Chiu et al. (2010) used ‘Graffiti’ as source of ‘*Pr*’ gene while we used ‘Sicilian purple’ and the both genotypes had differences in evolution, kind of major anthocyanins, and site of accumulation and also showed variation in content of anthocyanin. The ‘Graffiti’ was evolved as a natural mutant (Chiu et al., 2010) while Sicilian purple was evolved as an intermediate of Calabrese and cauliflower during its evolution process (Smith and King, 2000).

Further, anthocyanin biosynthesis in plants is regulated by transcriptional regulation of structural genes. MYB transcription factors constitute one of the major families of anthocyanin regulatory proteins which activate expression of anthocyanin structural genes as reviews in maize by Sainz et al. (1994). Regulatory genes were reported in different crops like *anthocyanin2* in *Petunia* flowers (Quattrocchio et al., 1999), *IbMYB1* gene in sweet potato tubers (Mano et al., 2007), *BoMYB2* in pre-floral apical meristematic region of purple cauliflower ‘Graffiti’ (Chiu and Li, 2012), and *RsUFGT* gene in radish roots (Muleke et al., 2017). During the present investigations, the expression of four *BoMYB* (*BoMYB1*, *BoMYB2*, *BoMYB3*, and *BoMYB4*) genes was studied, and we observed that expression of *BoMYB1* and *BoMYB2* was higher in ‘PC-1’ than the white curded ‘DC-466’ and bright purple curded variety ‘Graffiti’. *BoMYB3* and *BoMYB4* genes were found to be expressed at a very high level in Graffiti, however, *BoMYB3* was down regulated in PC-1 while the expression of *BoMYB4* was a bit higher in ‘PC-1’ compared to ‘DC-466’, but was substantially lower than ‘Graffiti’. These observations indicate that different modes of regulations of anthocyanin pathway are operational in ‘PC-1’ and ‘Graffiti’. Both the genotypes differ for their evolutionary process, kind, and concentration of anthocyanin and also for site of accumulation. In red cabbage, increased expression of *BoMYB2* was and reduced expression of *BoMYB3* was associated with enhanced anthocyanin production (Yuan et al., 2009) which appears to be partially true for ‘PC-1’.

## Conclusion

The ‘purple cauliflower-1’ (or PC-1) genotype, developed through recurrent selection, was found to be a promising colored cauliflower rich in anthocyanin. The study revealed that genetics of purple curds in Sicilian purple ‘PC-1’ appears to be governed by single semi-dominant gene similar to purple cauliflower ‘Graffiti’. However, the position of the Pr locus appears to be quite different as none of the markers from high resolution map of Pr locus reported earlier by Chiu et al. (2010) could produce similar results. It was revealed by monomorphic banding pattern in purple ‘PC-1’ and white ‘DC-466’ and variation in gene expression pattern in ‘PC-1’ and ‘Graffiti’. Hence, identification of markers is essential for early seeding stage detection of desirable plants in breeding materials, because the purple pigmentation on apical shoot and stem parts did not segregate with curd color. Interestingly, occurrence of broccoli type plants in F_2_ generation and non-segregation of F_2:3_ progeny of one such plant warrants for further studies.

## Data Availability Statement

All datasets for this study are included in the article/**Supplementary Material**.

## Author Contributions

SSi and PK conceived the concept. SSi developed and evaluated breeding material, analyzed data, and drafted manuscript. PK introduced ‘Sicilian purple’ and initiated recurrent selection for development of ‘PC-1’. RM supported phenotypic observation, performed anthocyanin determination and marker analysis. MM did qPCR expression analysis and drafted the relevant part. SI helped in anthocyanin determination. SSa carried out HPLC analysis. BT supported overall field experimentation. SSi, PK and MM read the manuscript and approved.

## Funding

The research was carried out in an in-house project and for that the funding for the research was received from ICAR-Indian Agricultural Research Institute, New Delhi.

## Conflict of Interest

The authors declare that the research was conducted in the absence of any commercial or financial relationships that could be construed as a potential conflict of interest.
