# Supplementary material for: Genetics and Expression Analysis of Anthocyanin Accumulation in Curd Portion of Sicilian Purple to Facilitate Biofortification of Indian Cauliflower
Source: Front Plant Sci. 2020 Jan 30;10:1766. doi: 10.3389/fpls.2019.01766 (PMC7003135; doi:10.3389/fpls.2019.01766)
Supplement: Supplementary file 1 [file DataSheet_1.docx]

**Supplementary Table 1**. **Oligonucleotide primers used for PCR analysis (Source: Yuan et al., 2009; Chiu et al., 2010)**

| **Primer** | **Sequences (5’-3’)** | **Annealing temperature (°C)** |
| --- | --- | --- |
| BoMYB1- F | AGTATGACATATTGTAACAAAGCTGGGGAGAAG | 54.5 |
| BoMYB1 -R | CGTACCTTTTGGAACGAGCCCGTCA | 57.9 |
| BoMYB2- R | GGAAACAGGTGGTCTTTAATTGCT | 50.7 |
| BoMYB2 -F | AGCTCAAATTTATCATCATCTTTGTTACATGTGATTA | 52.4 |
| BoMYB3 -F | GTCCAAACGGTTGAGAAAAGGTGCATAT | 54.4 |
| BoMYB3-R | CCAGCTCTTAAAGGAACTTGGTGCCATTTCCC | 58.8 |
| BoMYB4-F | TGAGTAAGAAACATGAACCAGGTTGT | 51.5 |
| BoMYB4-R | ATCCAAGGCATAGGGGAACAAAT | 51.6 |
| BoMYB2m R | GCTTGAGGAAAAGGGTTTATATTGTGGCT | 54.2 |
| BoMYB2m F | GTCGGAAGTGTTTAAAGAGAGAACATGAT | 51.9 |
| BoMYB2m R | GCTTGAGGAAAAGGGTTTATATTGTGGCT | 54.2 |
| BoMYB2m F | TTAGAGCTATCGGGGCGTGTCTAGAGA | 56.7 |
| BoMYB3m R | TGAAACTGAAGGAGTACTCCCTTGGTAAC | 54.4 |
| BoMYB3m F | GGTGCATGAAGCCGTTCTCTCTTTTAATC | 54.6 |
| BoMYB4m R | AAGGAAGGTTTCTCTTTTTCATTTGGGTCTTAC | 54 |
| BoMYB4m F | CCCAAGTGTATCAGCTACAGAAGAAGCCT | 54.2 |
| BoMYB4m R | AAGGAAGGTTTCTCTTTTTCATTTGGGTCTTAC | 54 |
| BoMYB4m F | TCGTTGTTGGTTTGTCGTTCAGTGAAAT | 56.2 |

**
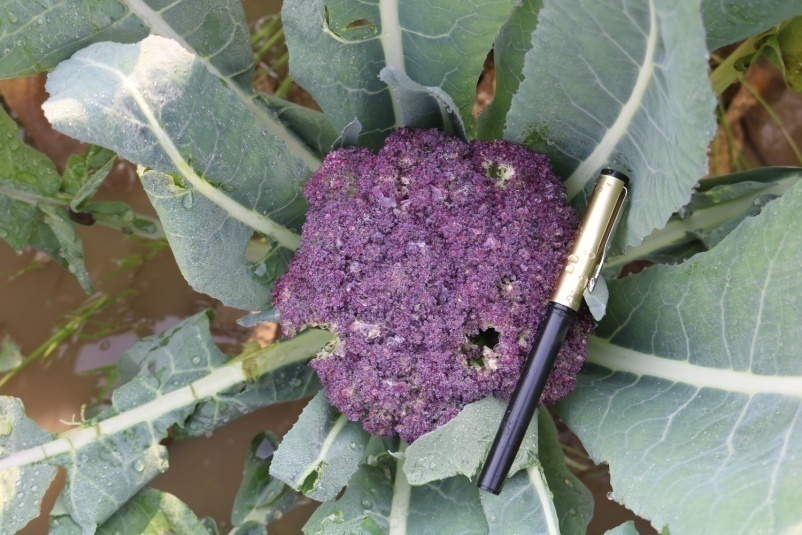

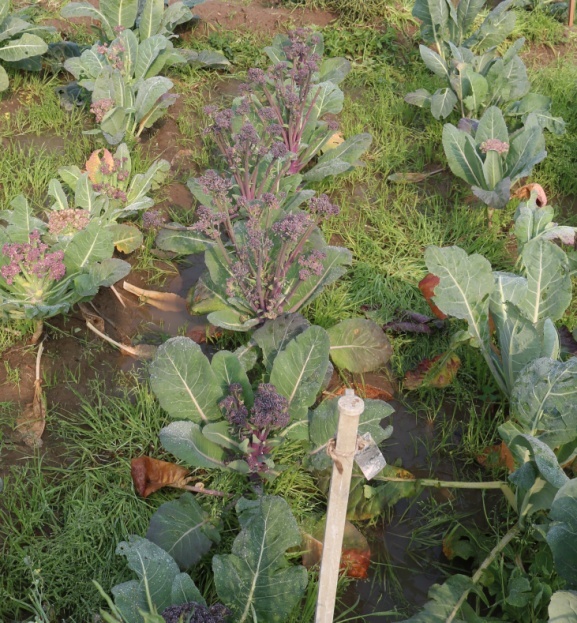
**

**Supplementary Figure 1(A-B) ǀ ‘Broccoli type’ plants in F_2_ (A), fixed line DC-466-1-76 in F_2:3_ (B)**


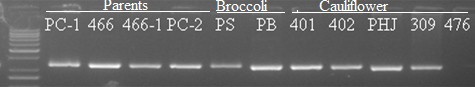


A. BoMYB2 (331 bp)


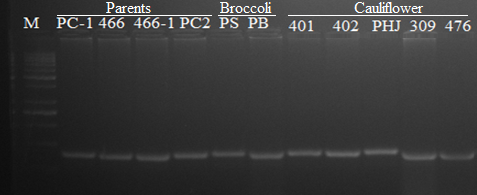


B. BoMYB1 (130 bp)


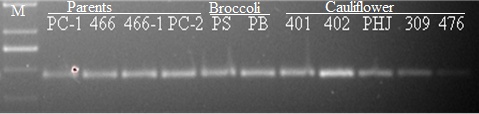


C. BoMYB2m (190 bp)


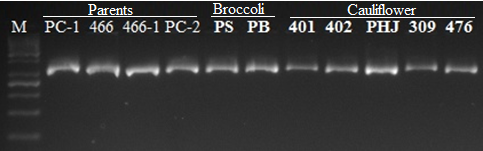


D. BoMYB3m (400 pb)


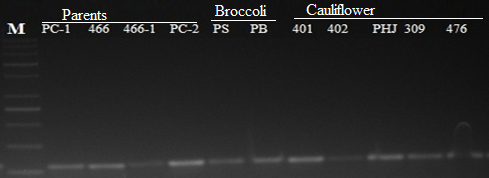


E. BoMYB4m (110 bp)

**Supplementary Figure 2A-E ǀ Amplification of molecular markers in parents and other genotypes.** BoMYB2 (A), BoMYB1 (B), BoMYB2m (C), BoMYB3m (D) and BoMYB4m (E) marker in genotypes. Here, M_50_- Marker 50 bp; Parents: PC-1, 466; Bulks: 466-1 and PC-2; Broccoli: PS (Palam Samridhi) and PB (Pusa Purple Broccoli); and Indian white cauliflower- 401 (Pusa Shukti), 402 (DC-402), PHJ (Pusa Himjyoti), 309 (Pusa Sharad) and 476 (Pusa Paushja).

**
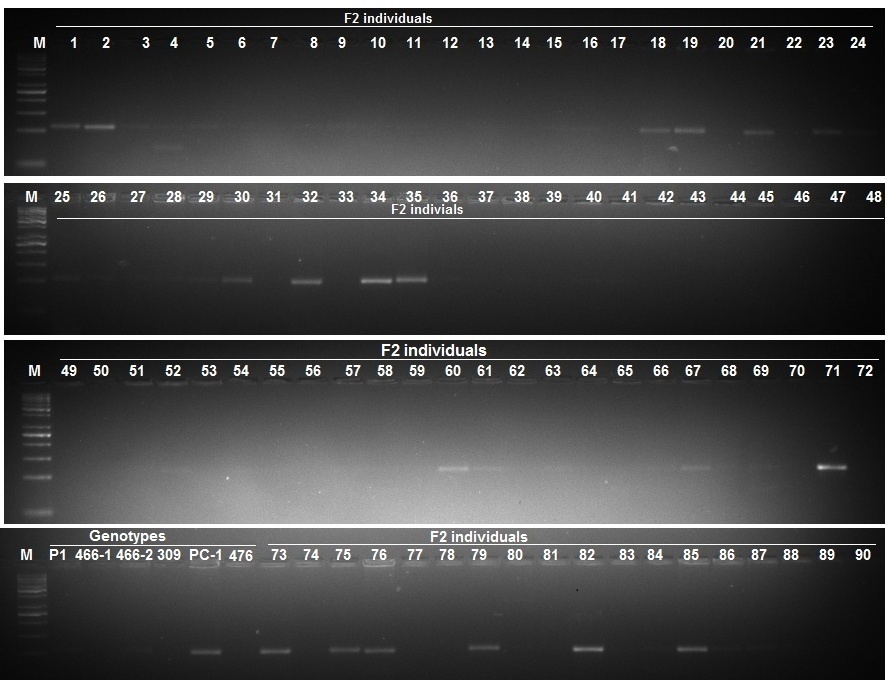
**

|  | **Phenotyping and genotyping of first 90 individuals of 173 F_2_ population** | | | | | | | | | | | | | | | | | | | | | | | | Total of phenotyping scoring | | Total of genotype scoring | |
| --- | --- | --- | --- | --- | --- | --- | --- | --- | --- | --- | --- | --- | --- | --- | --- | --- | --- | --- | --- | --- | --- | --- | --- | --- | --- | --- | --- | --- |
| Plant No. | 1 | 2 | 3 | 4 | 5 | 6 | 7 | 8 | 9 | 10 | 11 | 12 | 13 | 14 | 15 | 16 | 17 | 18 | 19 | 20 | 21 | 22 | 23 | 24 | Purple | White | Present | Absent |
| Phenotype | P | P | P | P | P | P | P | P | W | P | W | P | W | P | W | P | P | P | P | W | P | P | P | P | 19 | 5 | 9 | 15 |
| Genotype | + | + | + | + | + | - | - | - | - | - | - | - | - | - | - | - | - | + | + | - | + | - | + | - |  |  |  |  |
| Plant No. | 25 | 26 | 27 | 28 | 29 | 30 | 31 | 32 | 33 | 34 | 35 | 36 | 37 | 38 | 39 | 40 | 41 | 42 | 43 | 44 | 45 | 46 | 47 | 48 |  |  |  |  |
| Phenotype | P | W | P | P | P | P | P | P | P | P | P | P | P | W | W | W | P | P | P | P | P | W | P | W | 17 | 7 | 7 | 17 |
| Genotype | + | + | - | - | + | + | - | + | - | + | + | - | - | - | - | - | - | - | - | - | - | - | - | - |  |  |  |  |
| Plant No. | 49 | 50 | 51 | 52 | 53 | 54 | 55 | 56 | 57 | 58 | 59 | 60 | 61 | 62 | 63 | 64 | 65 | 66 | 67 | 68 | 69 | 70 | 71 | 72 |  |  |  |  |
| Phenotype | W | W | W | P | P | P | W | P | P | P | P | P | P | W | P | W | P | P | W | W | P | P | P | W | 15 | 9 | 7 | 17 |
| Genotype | - | - | - | + | - | - | - | - | - | - | - | + | + | - | + | - | - | - | + | - | + | - | + | - |  |  |  |  |
| Plant No | P1 | P1 | P1 | 309 | P2 | 476 | 73 | 74 | 75 | 76 | 77 | 78 | 79 | 80 | 81 | 82 | 83 | 84 | 85 | 86 | 87 | 88 | 89 | 90 |  |  |  |  |
| Phenotype | W | W | W | W | P | W | P | W | P | P | P | W | W | P | W | P | P | P | W | P | P | W | W | P | 13 | 5 | 7 | 11 |
| Genotype | - | - | - | - | + | - | + | - | + | + | - | - | + | - | - | + | - | - | + | + | - | - | - | - | **64** | **26** | **30** | **60** |

No. of F_2_ individuals observed here = 90; No. of recombinants = 41

**Supplementary Figure 3 ǀ** **Amplification of BoMYB3 marker in genotypes and 90 F_2_ plants**. M_50_- Marker 50 bp. White curd genotypes: DC-466 (Parent), DC-466-1, DC-466-2 and DC-309 (Pusa Sharad); purple curd genotypes: PC-1 (parent-2), 476 (Pusa Paushja)- a white curd genotype. S.No. 1 - 90 are F_2_ plants.
